# The Role and Antagonistic Effects of miR-16-5p in the Regulation of *ADP-Ribosylation Factor-Like Tumor Suppressor Gene 1* in Lung Cancer Cells

**DOI:** 10.5152/eurasianjmed.2023.23073

**Published:** 2023-10-01

**Authors:** Tuğba Nurcan Yüksel, Esra Bozgeyik, İbrahim Bozgeyik

**Affiliations:** 1 Department of Pharmacology, Tekirdağ Namik Kemal University Faculty of Medicine, Tekirdağ, Turkey; 2 Department of Medical Services and Techniques, Adiyaman University Vocational School of Health Sciences, Adiyaman, Turkey; 3 Department of Medical Biology, Adiyaman University Faculty of Medicine, Adiyaman, Turkey

**Keywords:** ARLTS1, ARL11, cancer, lung cancer, miR-16-5p

## Abstract

**Objective:**

*ADP-ribosylation factor-like tumor suppressor gene 1* is a member of the Ras superfamily of small guanosine triphosphatases that are known to be involved in multiple regulatory pathways in the multistage development of human cancers. Also, *ADP-ribosylation factor-like tumor suppressor gene 1* expression levels have been reported to be dramatically lower in both cancer cell lines and tumor tissues compared to controls. Accordingly, defects in the regulation of the *ADP-ribosylation factor-like tumor suppressor gene 1* gene seems have key tumor suppressive effects in the formation and development of human cancers including lung cancer. Moreover, microRNAs regulating the expression of *ADP-ribosylation factor-like tumor suppressor gene 1* have not been described previously. Accordingly, the present study aimed to reveal the influence of miR-16-5p on the regulation of *ADP-ribosylation factor-like tumor suppressor gene 1* gene.

**Materials and Methods:**

A549 lung adenocarcinoma cells were used. For the overexpression and silencing experiments of miR-16-5p synthetic microRNA mimics and inhibitors were used, respectively. Gene expression analyses were achieved with the help of quantitative real-time polymerase chain reaction.

**Results:**

MiR-16-5p was identified to be predictive target of *ADP-ribosylation factor-like tumor suppressor gene 1* and directly targets the expression of *ADP-ribosylation factor-like tumor suppressor gene 1* as revealed by the overexpression and silencing experiments. Specifically, it was found that miR-16-5p-overexpressed A549 cells showed a decrease in *ADP-ribosylation factor-like tumor suppressor gene 1* gene expression, whereas miR-16-5p-suppressed cells showed an increase in expression. These findings possibly suggest that miR-16-5p is the direct regulatory microRNA that posttranscriptionally regulates the expression of *ADP-ribosylation factor-like tumor suppressor gene 1*.

**Conclusion:**

Collectively, miR-16-5p seems to be a key regulatory molecule involved in the posttranscriptional regulation of the *ADP-ribosylation factor-like tumor suppressor gene 1*, and it might be responsible for the downregulation of this gene in lung cancer.

**Main Points**

- In this study, the interaction between miR-16 and the ARLTS1 gene was determined.
- miR-16-5p seems to be a key regulatory molecule in the regulation of ARLTS1 gene.
- miR-16-5p is possibly responsible for the downregulation of ARLTS1.

## Introduction

Lung cancer is the most common malignancy worldwide and the leading cause of cancer-related deaths in men and women. According to 2020 global cancer statistics, 2 206 771 (11.4%) new cases of lung cancer and 1 796 144 (18.0%) lung cancer-related deaths were estimated to occur.^1^ Treatment of lung cancer largely depends on the early diagnosis of the disease and its pathological stage. Therefore, the determination of biomarkers that can be used in the early diagnosis of lung cancer is important in the fight against cancer.

*ADP-ribosylation factor-like tumor suppressor gene 1* is a member of the ARF family, the Ras superfamily of small guanosine triphosphatases known to be involved in numerous regulatory pathways in the multistage developmental process of human cancers.^2^ Structural and functional disorders caused by the *ARLTS1* gene in cancer are of 3 types: genomic loss, gene polymorphisms, and epigenetic changes. The main reason for downregulation of *ARLTS1* appears to be promoter methylation.^3^ In addition, *ARLTS1* expression levels have been reported to be dramatically lower in both cancer cell lines and tumor tissues compared to controls. Accordingly, defects in the regulation of the *ARLTS1* seem to be a key factor in the formation and development of human cancers.^2,4^

Previously, it was assumed that only oncogenes and tumor suppressor genes were involved in the molecular basis of cancer. However, with the discovery of noncoding RNA (ncRNA) molecules, which play significant regulatory roles in regulating gene expression, this assumption has largely lost its validity.^5^ The studies conducted in the following years allowed the identification of numerous classes of noncoding RNAs, in particular long noncoding RNAs (lncRNAs) and microRNAs (miRNAs), which are involved in the regulation of gene expression and exhibit oncogenic/tumor suppressive properties.^6^ In particular, miRNAs have attracted great interest among noncoding RNAs because they are more stable and more easily targeted therapeutically.^7^ MicroRNAs are small noncoding RNAs about 18-22 nucleotides long.^8^ MicroRN induce posttranscriptional repression of their target genes by showing partial complementarity to the UTR (untranslated region) of their target mRNAs. The production of mature RNA sequences in the cell takes place through a series of processing events.^5^ Recent studies have shown that miRNAs mediate various cellular processes, such as cell cycle control and regulation of cell death mechanisms, through various targets.^6^

MiR-16, which has significant regulatory roles in the development and progression of human cancers, is localized in the intronic region of the Deleted in Lymphocytic Leukemia 2 (DLEU2) lncRNA gene mapped in the 13q14.2 chromosomal region.^9^ The miR-16 has been shown to negatively regulate and antagonize B cell lymphoma 2 (Bcl-2) to induce apoptosis.^10^ It has been shown that miR-16 regulates the expression of cyclin D1 (CCND1), cyclin D3 (CCND3), and cyclin E1 (CCNE1) to induce G0/G1 cell cycle arrest.^9^ Accumulating evidence also suggests that the expression level of miR-16 is low in the tumor tissues and lung cancer cells of non-small-cell lung cancer patients.^11,12^

Moreover, miRNAs regulating *ARLTS1* expression have not been described previously. Accordingly, the present study aimed to uncover the impact of miR-16-5p on *ARLTS1* regulation. To uncover the effects of miR-16-5p on *ARLTS1* regulation, overexpression/silencing experiments were performed with synthetic miRNA mimics/inhibitors in A549 lung cancer cells.

## Materials and Methods

### Cell Culture

In the study, A549 (CCL-185) lung cancer cells were obtained from ATCC (Manassas, Va, USA). Cells were cultured in Dulbecco’s modified Eagle’s medium (DMEM; Merck KGaA, Darmstadt, Germany) medium containing 10% fetal bovine serum (Merck KGaA, Darmstadt, Germany) and 50 U/mL penicillin/streptomycin (Merck KGaA, Darmstadt, Germany), in a carbon dioxide incubator with 95% air, 5% CO_2_, and 37°C.

### miR-16 Mimic and Inhibitor Transfections

Mimic and inhibitor experiments were performed to overexpress and suppress miR-16-5p in A549 cells. miR-16-5p mimics, inhibitors, and a negative control, which have no detectable target, were commercially obtained (Qiagen, Germany). For the transfection experiments, cells were seeded into 12-well plates at a concentration of 5 × 10^4^ cells/well. For the transfections, HiPerFect Transfection Reagent (Qiagen, Hilden, Germany) were used. The following transfection mixture was prepared: 100 µL DMEM, 4 µL HiPerFect Transfection Reagent (Qiagen, Germany), and 0.5 µL mimic or 4 µL inhibitor. The prepared mixture was incubated at room temperature for 30 minutes and delivered to cells dropwise and 10% fetal calf serum was added after about 4 hours. Following 72 hours post transfection, cells were removed for total RNA isolation.

### RNA Extractions and cDNA Synthesis

Following transfections, cells were collected and total RNA was extracted with the help of the miRNeasy Mini Kit (Qiagen, Germany). Cells were first lysed using Qiazol reagent, and recommendations of the manufacturer were followed. After the isolations, the RNA concentrations were measured using a NanoDrop 2000. RNA concentrations were approximately 100-1000 ng/µL and further adjusted to 500 ng/µL. For the synthesis of complementary DNA (cDNA), the miScript II RT kit (QIAGEN, Germany) was used. HiFlex buffer was used for cDNA synthesis of long RNAs. For the synthesis, 500 ng/µL RNA, 4 µL HiFlex buffer, 2 µL Nuclei Mix, 2 µL Reverse Transcriptase, and nuclease-free water up to 20 µL were used. The prepared mixture was incubated at 37°C for 60 minutes and 95°C for 5 minutes, and after the reaction, it was immediately placed on ice and stored at –80°C.

### Quantitative Expression nalysis by Real-Time Polymerase Chain Reaction

MiScript SYBR Green PCR Kit (Qiagen, Germany) and miR-16 primer assay kit were used to determine miR-16 expression levels. RNU6 was used as the reference gene to determine the expression level of miR-16. The reaction mixture prepared was subjected to thermal conditions at 94 °C for 15 minutes, 40 cycles at 94 °C for 15 seconds, at 55 °C for 30 seconds, and at 70 °C for 30 seconds. For the determination of ARLTS1 gene expression levels, gene-specific primers (F primer: 5’-CCCATCTCGCTCTTGGGTCAT-3’; R primer: 5’-CCACTGCTGAATCCTCTGTGTT-3’) and RealQ Plus 2x Master Mix (Ampliqon PCR Enzymes & Reagents, Denmark) were used. GAPDH (F primer: 5’-GATCATCAGCAATGCCTCCT-3’; R primer: 5’-TGTGGTCATGAGTCCTTCCA-3’) was used as a reference to determine the expression level of ARLTS1. The prepared reaction mixtures were incubated at following thermal conditions; 95 °C for 15 minutes, 40 cycles at 95 °C for 15 seconds, at 60 °C for 30 seconds, at 72 °C for 30 seconds. Cycle threshold values were calculated at the appropriate threshold value following all reactions. All reactions were repeated 3 times. 

### Statistical Analyses

All statistical evaluations were performed using GraphPad Prism, Version 8. For the calculation of gene expression levels of miR-16 and *ARLTS1*, 2^–ΔCt^ (ΔCt = Ct Target Gene – Ct Reference Gene) formula was used. Student’s *t*-test was used for comparisons between variables. For all results, those with *P* <.05 were considered significant. 

## Results

### Determination of miRNA Target by Using miRNA-Target Prediction Algorithms

**Figure 1.**
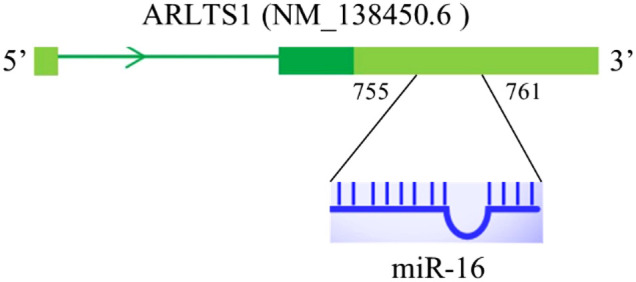
Complementary base-pairing between the *ARLTS1* and miR-16-5p. At positions 755-761 in the 3’-UTR region of the *ARLTS1*, miR-16-5p shows 7mer-m8 complementarity.

To determine the miRNAs posttranscriptionally targeting *ARLTS1* gene TargetScan^13^ miRNA-target prediction algorithm was used and miR-16-5p was selected. The most important criterion in determining the relevant miRNA was that the selected miRNA was not previously reported to be associated with *ARLTS1* in the literature. In the TargetScan database, it was determined that *ARLTS1* showed 7mer-m8 complementarity with miR-16-5p between positions 755-761 in the 3’-UTR region. As a result, it was determined that miR-16-5p could be a chief regulator of *ARLTS1* (Figure 1).

### Overexpression and Silencing of miRNA-16-5p

**Figure 2.**
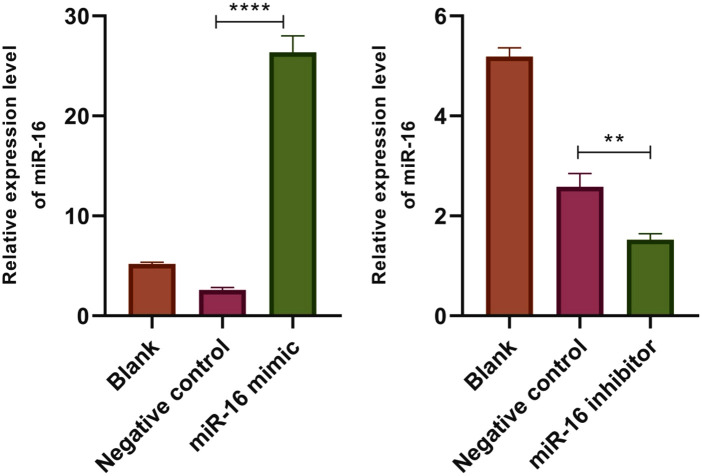
Ectopic expression levels of miR-16-5p in A549 lung cancer cells after miR-16-5p mimic (left) and inhibitor (right) transfections. ** *P *<.01; **** *P* <.0001.

To further explore the interaction between *ARLTS1* and miRNA-16-5p, overexpression and silencing experiments were performed with synthetic miRNA mimics and inhibitors, respectively, in A549 lung cancer cell lines. After miR-16-5p mimic and inhibitor transfections, miR-16-5p level was determined by quantitative polymerase chain reaction. As a result, it was found that the expression level of miR-16-5p increased approximately 10 times in the mimic transfected group compared to the negative control (*P* <.0001) and decreased approximately 1.6 times in the inhibitor transfected group compared to the negative control (*P* = .0032) (Figure 2). These results were found to be statistically significant.

### ARLTS1 Expression Levels Following Mimic/Inhibitor Transfections

**Figure 3.**
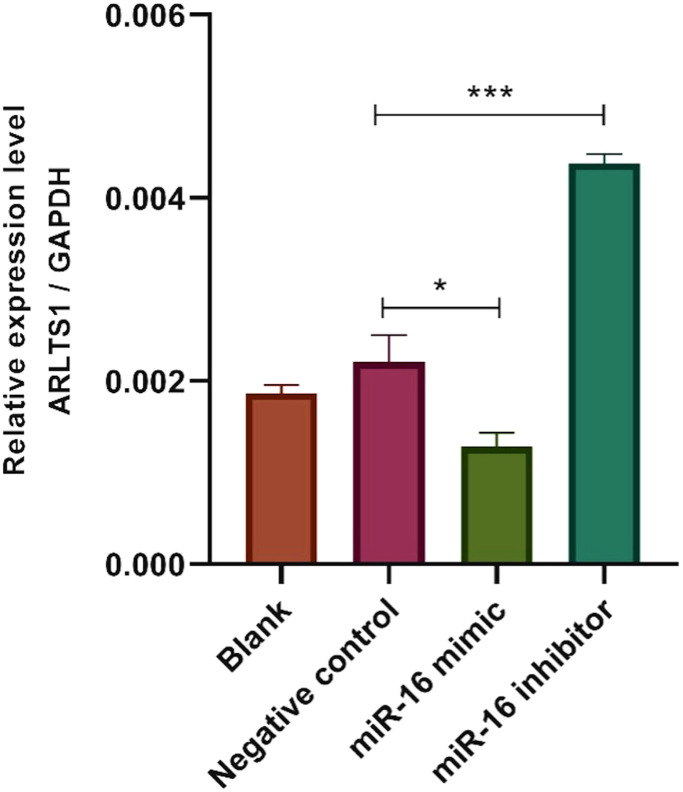
Differential expression levels of *ARLTS1* in A549 lung cancer cells treated with the miR-16-5p mimic and inhibitor transfections. *P <.05, ***P <.001.

To examine the impact of overexpression and silencing of miRNA-16-5p on *ARLTS1*, expression levels of *ARLTS1* were determined. As a result, *ARLTS1* expression level was found to be significantly decreased by approximately 1.7 times in miR-16-5p overexpressing cells (*P* = .0107). In contrast, the expression level of *ARLTS1* increased approximately 2-fold in cells in which miR-16-5p inhibitor was applied and miR-16-5p expression levels were suppressed (*P* = .0002) (Figure 3).

## Discussion

In this study, first, the interaction between miR-16 and the *ARLTS1* was determined by bioinformatics tools, and then overexpression and suppression experiments were performed in A549 lung cancer cells after miR-16 mimic and inhibitor transfections. Notably, it was found that the expression level of the *ARLTS1* decreased in miR-16-5p-overexpressed A549 cells and increased in miR-16-5p-suppressed cells, strongly suggesting that miR-16-5p is chief regulatory miRNA that posttranscriptionally fine-tune the expression of *ARLTS1*. Overall, we report here for the first time an as yet unidentified novel mechanism for posttranscriptional regulation of *ARLTS1*. miR-16-5p functions as an antagonistic molecule to silence the expression of *ARLTS1*, indicating that it might be used to modulate expression and functions of *ARLTS1*. 

Although limited, a few studies suggest that *ARLTS1* expression is downregulated in various types of human malignant disease, including prostate cancer,^14^ lung carcinomas, B cell chronic lymphocytic leukemia, ovarian carcinomas, and ovarian and breast cancer cell lines.^4^ The limited number of studies demonstrating *ARLTS1* expression makes it difficult to comment further on this issue. In addition, findings of the significant number of studies strongly indicate that genomic variations of *ARLTS1* is associated with the increased risk of breast cancer,^15,16^ multiple melanomas,^17^ colorectal cancer,^18^ prostate cancer,^19^ and ovarian cancer.^20^ In contrast to these studies, an accumulating body of evidence also suggests that there is no association between *ARLTS1* variants and chronic lymphocytic leukemia,^21^ colorectal cancer,^22^ basal cell carcinoma of the skin,^23^ sporadic breast cancer, prostate cancer, melanoma, thyroid papillary cancer, or laryngeal cancer.^24^

Furthermore, Yendamuri et al showed that overexpression of the *ARLTS1* in lung cancer cells reduced cell proliferation, stimulated apoptosis, and reduced the tumorigenic effect *in vivo.* ^25^ Particularly, in 9 lung cancer cell lines and 27 pairs of normal and lung cancer tissues, *ARLTS1* has been shown to be downregulated in all lung cancer cell lines and in approximately 37% of lung tumors compared to normal tissues.^25^ *ADP-ribosylation factor-like tumor suppressor gene 1* was also shown to be associated with the inhibition of cell proliferation in ovarian and breast cancers.^4^ These data show that *ARLTS1* plays a tumor suppressor role in the formation and progression of common malignancies. However, there are no studies on miRNAs targeting the *ARLTS1*. On the other hand, miR-16-5p has been shown to be poorly expressed in many cancer types. It has been shown that the expression level of miR-16-5p is low in the serum samples of non-small-cell lung cancer patients, and overexpression of miR-16-5p suppresses the proliferation of lung cancer cells and reduces cell migration and invasion.^26-28^

## Conclusion

In conclusion, the present findings identify miR-16-5p as a key regulatory molecule involved in the posttranscriptional regulation of the *ARLTS1*. The present study also suggests that deregulation of miR-16-5p might be responsible for the downregulation of *ARLTS1* in lung cancer. However, the current study has several limitations. Future more comprehensive studies are needed to fully decipher the functions and fate of the *ARLTS1* in human cancers. Particularly, gain-of-function and/or loss-of-function experiments of *ARLTS1* together with the mimic/inhibitor manipulations will be more valuable to better understand the miR-16-5p/ARLTS1 axis in lung cancer. Also, the use of additional lung cancer cell lines and normal lung epithelial cells will be more valuable to fully comprehend more accurate results. Additionally, a luciferase reporter assay will be more valuable to evaluate the direct interaction between miR-16-5p and the ARLTS1 gene. Also, further experiments with tumor xenograft models and/or genetically or chemically engineered animal models of lung cancer will be more valuable to understand better about the miR-16-5p-mediated regulation of ARLTS1.
